# The effect of EEG and fNIRS in the digital assessment and digital therapy of Alzheimer’s disease: a systematic review

**DOI:** 10.3389/fnins.2023.1269359

**Published:** 2023-11-21

**Authors:** Yucheng Zhang, Yue Zhang, Zhouhao Jiang, Mengxue Xu, Kunqiang Qing

**Affiliations:** ^1^Department of Mathematics, College of Natural Sciences, University of Texas at Austin, Austin, TX, United States; ^2^Department of Psychology, School of Psychology, Shenzhen University, Shenzhen, China; ^3^Department of Computer Control and Automation, Nanyang Technological University, Singapore, Singapore; ^4^Department of Neurology, Chongqing Public Healthy Medical Center, Chongqing, China; ^5^Research Group of Brain-computer Interface, Brainup Institute of Science and Technology, Chongqing, China

**Keywords:** Alzheimer’s disease, EEG, fNIRS, digital assessment, digital therapy

## Abstract

In the context of population aging, the growing problem of Alzheimer’s disease (AD) poses a great challenge to mankind. Although there has been considerable progress in exploring the etiology of AD, i.e., the important role of amyloid plaques and neurofibrillary tangles in the progression of AD has been widely accepted by the scientific community, traditional treatment and monitoring modalities have significant limitations. Therefore novel evaluation and treatment modalities for Alzheimer’s disease are called for emergence. In this research, we sought to review the effectiveness of digital treatment based on monitoring using functional near-infrared spectroscopy (fNIRS) and electroencephalography (EEG). This work searched four electronic databases using a keyword approach and focused on journals focusing on AD and geriatric cognition. Finally, 21 articles were included. The progress of digital therapy and outcome monitoring in AD was reviewed, including digital therapy approaches on different platforms and different neuromonitoring techniques. Because biomarkers such as theta coherence, alpha and beta rhythms, and oxyhemoglobin are effective in monitoring the cognitive level of AD patients, and thus the efficacy of digital therapies, this review particularly focuses on the biomarker validation results of digital therapies. The results show that digital treatment based on biomarker monitoring has good effectiveness. And the effectiveness is reflected in the numerical changes of biomarker indicators monitored by EEG and fNIRS before and after digital treatment. Increases or decreases in the values of these indicators collectively point to improvements in cognitive function (mostly moderate to large effect sizes). The study is the first to examine the state of digital therapy in AD from the perspective of multimodal monitoring, which broadens the research perspective on the effectiveness of AD and gives clinical therapists a “reference list” of treatment options. They can select a specific protocol from this “reference list” in order to tailor digital therapy to the needs of individual patients.

## 1 Introduction

Currently, AD is considered a neurodegenerative disorder in which the two main pathological features are senile plaques and neurofibrillary tangles. Senile plaques consist of a central core of β-amyloid proteins with structural degeneration of the surrounding neurons or synapses, while the aberrant buildup of hyperphosphorylated Tau proteins in the perinuclear cytoplasm of neurons results in neurofibrillary tangles (Perl, 2010). Among the various diseases in developed populations, AD consumes the most social resources (Meek et al., 1998; Bonin-Guillaume et al., 2005) but its true etiology remains unclear (Burns and Iliffe, 2009). The current hypotheses explaining the etiology of AD include the β-amyloid hypothesis, the Tau protein hypothesis, the cholestatic hypothesis, and some other hypotheses, among which the most dominant hypothesis is the β-amyloid hypothesis. This hypothesis suggests that the cause of AD may lie in the accumulation of β-amyloid (Aβ) in the brain (Hardy and Allsop, 1991; Mudher and Lovestone, 2002), which has been supported by studies on patients with Down syndrome (Lott and Head, 2005; Nistor et al., 2007) and studies on genetically cloned mice (Games et al., 1995; Hsiao et al., 1996; Masliah et al., 1996; Lalonde et al., 2002).

One of the precursor symptoms of AD is mild cognitive impairment (MCI), a cognitive impairment that falls somewhere between a healthy state and AD (Grundman et al., 2004). Although the disorder does not significantly affect daily life, people with mild cognitive impairment have experienced one or more declines in cognitive function (Petersen et al., 1999). It is important to note that not all MCI will eventually evolve into AD. MCI can be divided into two subtypes, amnestic MCI (aMCI) and non-amnestic MCI (naMCI). The main manifestation of aMCI is a significant decline in memory function, while the main manifestation of naMCI is a decline in other cognitive functions, such as visuospatial ability and attention (Grundman et al., 2004; Csukly et al., 2016). Among them, aMCI has a higher risk of developing into AD. From the perspective of neurobiology, there is no recognized answer to the question of how healthy elderly people develop into MCI and eventually into AD. But there are two mainstream hypotheses that try to describe this process and the reasons behind it. One of the mainstream hypotheses is β-amyloidosis. This hypothesis can be further subdivided into amyloid β-protein sedimentary plaque pathogenic hypothesis and amyloid β-protein oligomer pathogenic hypothesis. Amyloid β-protein’s hypothesis of sedimentary plaque pathogenicity holds that the structure and function of amyloid β-protein’s precursor protein change with gene mutation, which leads to a long-term high concentration of amyloid β-protein in cerebrospinal fluid, and finally forms plaque deposits after the dual effects of physical sedimentation and protein coagulation, which hinder the normal operation of the nervous system (Mullan et al., 1992; Howland et al., 1995; Yin et al., 2017). Amyloid β-protein’s oligomer pathogenic hypothesis holds that Aβ oligomer binds to the receptor of nerve cells, which changes the structure of synapses, thus destroying the communication between neurons (Lacor et al., 2007). Another mainstream hypothesis is the Tau protein hypothesis. Tau protein hypothesis holds that the chief culprit that leads an elderly person to develop from a healthy state to MCI and eventually to AD is the abnormality of Tau protein (Mudher and Lovestone, 2002). When Tau protein is hyperphosphorylated, it will be paired and combined with other Tau proteins. The product of pairing and combination is neurofibrillary tangles (Goedert et al., 1991). This entanglement will destroy the microtubules in nerve cells and eventually lead to the collapse of nerve transport system (Iqbal et al., 2005). Without a normal transportation system, the information transmission between nerve cells cannot be carried out normally, which eventually leads to the death of nerve cells (Chun and Johnson, 2007).

Alzheimer’s disease faces varying degrees of challenges in treatment and monitoring due to the lack of clarity on the etiology. Challenges in the treatment of AD include the lack of clarity on the cause, the limited effectiveness of treatment, and the high failure rate of drug development. The etiology of AD is still only a theory in human knowledge, and the pathogenesis has not been truly understood (Long and Holtzman, 2019). Probably due to the limited knowledge of the etiology, most of the current treatments can only provide symptomatic relief, but not yet achieve full recovery. The lack of understanding of the etiology of AD may also pose difficulties in drug development, as the success rate of current drugs for AD is much lower than the failure rate, and they are often not marketed due to poor efficacy and other problems in clinical trials (Doody et al., 2014). The main challenges in monitoring AD are the lack of exact biomarkers and the high subjective factor of monitoring. Throughout the process, physicians lack accurate and effective biomarkers to monitor the disease, both for the initial diagnosis and for the final assessment of intervention effects (Blennow et al., 2015). Therefore, physicians have to rely on some subjective knowledge or experience to make judgments, which happens to introduce the interference of subjective factors (Canevelli et al., 2013).

Digital cognitive assessment is a method of assessing cognitive function using digital technology, and the vehicle for the assessment tool is often a digital device such as a computer or tablet. The advent of digital cognitive assessment has somewhat compensated for the limitations of conventional testing with paper and a pencil in the assessment of AD. Most traditional paper-and-pencil tests can only detect obvious symptoms in clinical populations and are not sensitive enough to subtle preclinical impairments (Rentz et al., 2013). This could result in a reduced correlation between the outcomes of paper-and-pencil tests and AD biomarkers (especially cross-sectional relationships) in clinically normal older adults (Duke Han et al., 2017). Digital cognitive assessment, on the other hand, may provide a more accurate record of data such as reaction time, thereby improving sensitivity to symptom identification (Zygouris and Tsolaki, 2015). Digital cognitive assessment has other advantages, such as the ability of the computer to automatically generate different alternative versions of the same test, thus reducing the practice effect seen in paper-and-pencil tests (Miller and Barr, 2017); the portability of mobile devices facilitates the increase in the number of information collections, thus facilitating longitudinal studies and enhancing the richness and reliability of longitudinal data (Sliwinski et al., 2018); digital cognitive assessments, facilitated by computerized assessors, offer superior standardization and objectivity compared to traditional paper-and-pencil tests with human assessors. This digital approach reduces the impact of subjective judgments and enhances assessment accuracy, thereby improving the overall evaluation process (Wild et al., 2008). In conclusion, the advent of digital cognitive assessment is a boon to patients with AD as well as researchers and clinical workers, and it is necessary to develop more effective digital cognitive assessment tools.

Pharmacological treatment for MCI and AD includes cholinesterase inhibitors and NMDA receptor antagonists. Cholinesterase inhibitors improve cognitive function (Birks, 2006), while the effectiveness of NMDA receptor antagonists for AD is uncertain (Reisberg et al., 2003; Tariot et al., 2004; Schneider et al., 2011; Howard et al., 2012). Medication only addresses symptoms and has side effects such as nausea, vomiting, headache, insomnia, and muscle cramps (Birks, 2006). The need for research on new cognitive therapies is emphasized. Non-pharmacological brain neuromodulation techniques are being explored to overcome treatment limitations (Cai et al., 2019) Transcranial direct current stimulation (tDCS) shows potential in enhancing cognition but may have side effects like headache and skin irritation. Other techniques like tACS (Liu et al., 2023) and TMS (Menardi et al., 2022) are investigated but their effectiveness is debated. Digital cognitive therapy utilizing digital technologies shows promise in treating early AD and MCI. Personalized treatment plans, on-demand learning tasks, and virtual reality exercises benefit patients (Allison et al., 2016). Digital therapy has fewer side effects compared to medication. Combining games with BCI technology enhances attention and cognitive abilities in individuals with MCI (Hadjiaros et al., 2023). Digital therapy offers flexibility and safety. Patients can remotely engage in training activities with real-time data tracking. Self-medication before seeking medical attention is possible for suspected Alzheimer’s, facilitating early treatment and potential cost savings (Fantozzi et al., 2022).

Invasive nerve detection techniques involve minimally invasive procedures to record and control neural signals. Sander’s study used invasive methods, specifically lumbar puncture, to obtain biomarkers and determine amyloid load levels for detecting AD (Verfaillie et al., 2019). Intracranial EEG acquisition involves inserting a fine electrode into the brain to record deep brain activity with higher spatial resolution than cortical EEG (Buzsáki et al., 2017). Invasive techniques offer increased accuracy and stability compared to non-invasive methods but carry potential risks like infection due to the need for surgical procedures. Non-invasive neurological detection techniques, such as EEG and fNIRS, enable the recording of neural signals using external sensors. These techniques have advantages over traditional invasive methods, such as cerebrospinal fluid testing, including being non-invasive, reproducible, and avoiding risks and discomfort during sampling. EEG measures potential changes on the scalp in real time to monitor abnormal neuron loss and activity changes associated with AD, allowing for early diagnosis. FNIRS utilizes near-infrared light to infer neural activity and blood oxygen levels, providing biomarkers that can be used for monitoring AD perfusion abnormalities (Huppert et al., 2009). EEG and fNIRS are portable, relatively inexpensive, and easy to operate, and the new generation of monitoring systems is wireless and battery-powered, resembling the size of a smartphone. Multimodal neuroimaging techniques have also become prominent in digital AD treatment by offering more patient information and compensating for the limitations of single-modal measurements (Teo et al., 2016). Overall, replacing invasive techniques with non-invasive ones for biomarker acquisition holds significant research value in the field of AD.

Currently, EEG and fNIRS have a wide range of applications in AD research. More studies have focused on the use of EEG to monitor the EEG activity of AD patients during cognitive training, and to investigate the effects on cognitive function by observing the characteristic changes in brain regions in different frequency bands (Anguera et al., 2013; Abiri et al., 2019; Ata et al., 2019). FNIRS has also been used as an emerging tool to study the changes in cerebral blood flow and oxygenation level of AD patients during dance video training, as well as its effects on cognition and emotion (Doi et al., 2013; Acevedo et al., 2022). In addition, there are also multimodal studies combining EEG with fNIRS to obtain more precise physiological information (Angsuwatanakul et al., 2020; Cicalese et al., 2020; Perpetuini et al., 2020). Moreover, it is also possible to improve training modalities through neurofeedback (Nan et al., 2012; Acevedo et al., 2022). Although, there are current challenges in game design, paradigm selection, and data processing, these studies contribute to the development of innovative therapeutic modalities.

In this review, our goal is to systematically review the current status of digital therapies regarding AD and describe the relationship between the effects of these digital therapies and biomarkers. In addition, we will critically discuss the drawbacks, potential, and future of digital treatments, EEG, and fNIRS in the treatment and monitoring of AD. We discuss digital therapy in two categories based on technology platforms: (i) Tablet and laptop-based, (ii) Novel system (eg. VR, Dance Video Games), and place digital therapy in three monitoring contexts, EEG, fNIRS, and hybrid devices consisting of EEG and fNIRS, respectively, to discuss the association between treatment effects and biomarkers. The association between treatment effects and biomarkers was discussed in three monitoring contexts: EEG, fNIRS, and hybrid devices. For each digital treatment, validation is discussed based on biomarkers.

## 2 Methods

### 2.1 Search strategies

From March 2023 to July 2023, We searched the relevant literature using several searchers and databases such as Google Scholar, Web of Science and Pubmed. Combinations of the following keywords were used: “digital therapy,” “game training,” “Alzheimer’s disease (AD),” “MCI,” “EEG,” “fNIRS,” and “the elder.” Subsequently, we conducted a second search by narrowing the keywords identified in the first search around the study topic again. In addition, we searched journals specifically focused on AD as well as neurological aspects to find relevant information: Journal of Alzheimer’s Disease, Alzheimer’s & Dementia, Frontiers in Aging Neuroscience, and Neurobiology of Aging.

### 2.2 Inclusion and exclusion criteria

Published papers were selected if they involved digital therapy of the elderly and monitoring with EEG or fNIRS. We excluded studies that used other techniques (such as fMRI) for monitoring, did not include older adult. We also excluded studies that used non-numeric cognitive training. This is because the studies focused on investigating the effectiveness of numerical cognitive training methods. The exclusion of non-numeric methods helps to maintain a clear and specific scope of the studies, making the objectives and findings clearer and more focused. And it is important to note that ensuring methodological consistency helps to avoid the introduction of variability due to differences in training methods, materials, or settings.

### 2.3 Procedures

**Figure 1:**
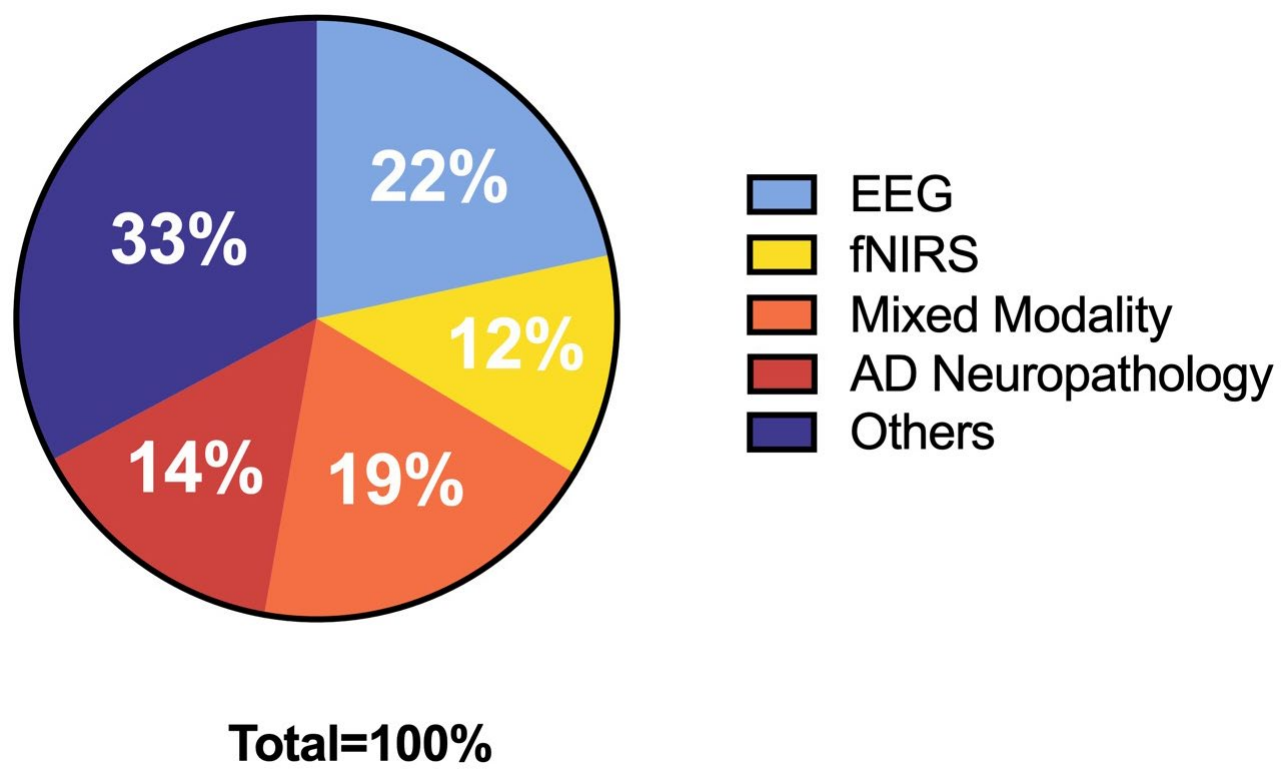
Pie chart of the structure of selected references.

**Figure 2:**
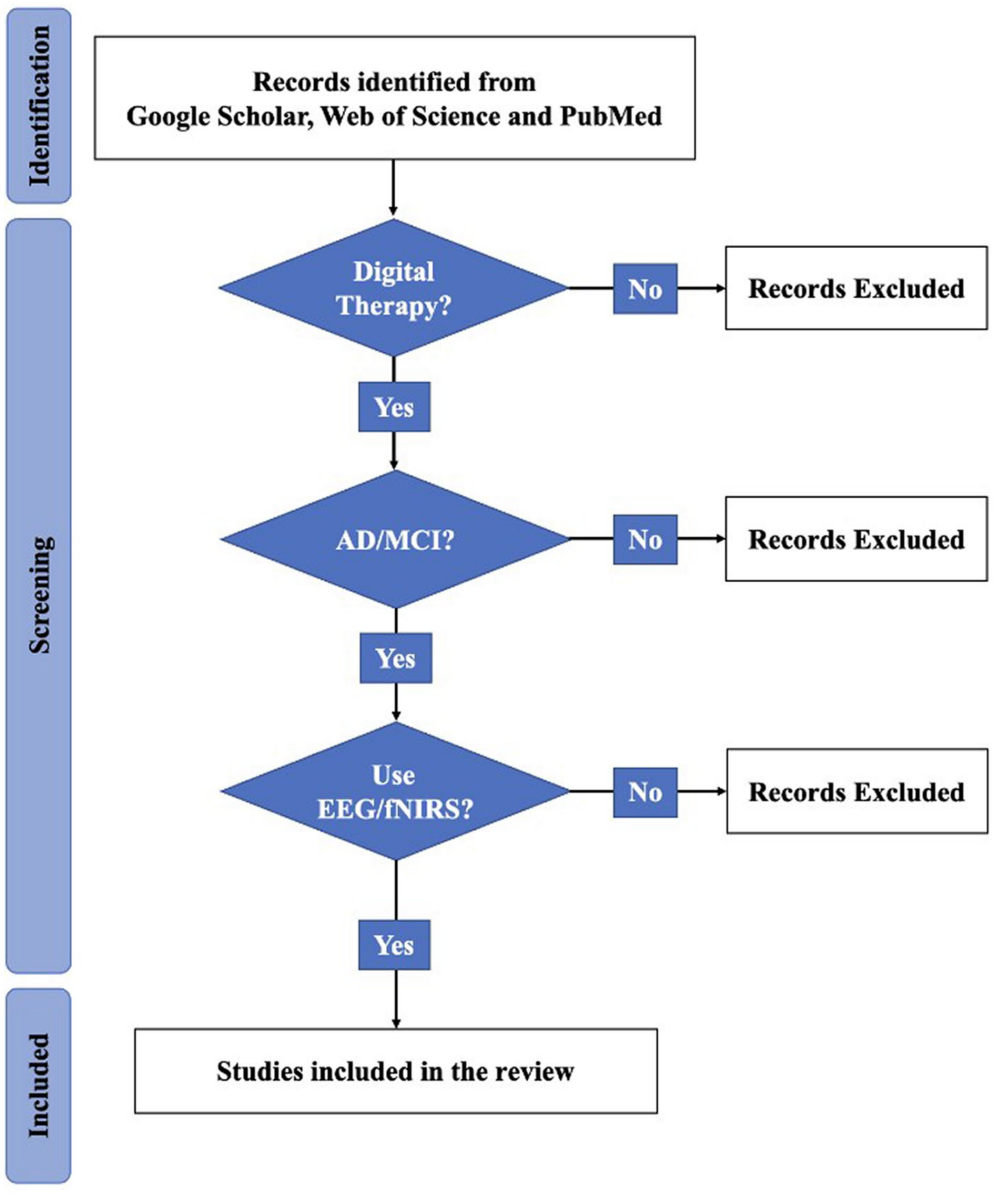
Flow diagram.

While categorizing and counting the collected literature, we found that the rest of the databases are covered by Google Scholar. And, he also has a wider range of literature resources. So, we only chose Google Scholar in order to avoid duplication of information when mapping. Using Google Scholar, a total of 41,800 pieces of literature were screened. Based on the keywords mentioned in the search strategy and the necessary relevant background, we finally cited 129 references. The specific categories and number of references are shown in Figure 1. Among the selected references, there are 27 references introduce EEG, 15 in the direction of fNIRS, 24 in mixed modality, 18 in AD neuropathology and 41 with information about other contexts. During the dialectical analysis, we further filtered the articles, and ended up with a total of 21 articles. The number of screened 21 articles about EEG, fNIRS, EEG + fNIRS are 10,7,4, respectively. Subsequently, we extracted different keywords according to the content of the articles and organized the literature, the details of which are shown in Table 1. Seventeen additional articles were added to the review based on the original literature search due to the limited elaboration of the limitations of digital assessment and neuromonitoring modalities in the originally collected literature, which limited the summary of the limitations of digital therapy combined with neuromonitoring. Figure 2 depicts the flow diagram.

**Table 1:** Digital therapy based on EEG/fNIRS/EEG + fNIRS for AD/MCI.

	Authors	Instrument	Longitudinal/cross-sectional	Platform	Channels	Feature extraction	Validation	Participants (n)	Effect size
EEG	Anguera et al.	3D video game (NeuroRacer)	Longitudinal (6 months)	laptop	5(Fz, FPz, AF3, AF4, AFz)	mft power	Significant increase in mft power after the multitask mission	174	Medium to large (d: 0.50–1.0)
	Jirayucharoensak et al.	NFT	Longitudinal (3 months)	computer	4(AF3, AF4, O1, O2)	Beta and Alpha bands	\	119	\
	Thapa et al.	VR intervention program	Longitudinal (8 weeks)	VR	19	TBR TAR DAR	Theta was lower around the parietal and temporal regions. TBR is reduced in temporal and parietal regions	68	TBR: temporal (*p* = 0.035), parietal (*p* = 0.027)
	Rose et al.	VR	Longitudinal (1 month)	computer	30	N300, P3, PP, AUC	The amplitude of the late positive complex increases in the right frontal electrode cluster, whereas both were decreased on the left side	58	*p* < 0.005
	Abdessalem et al.	VR Train, Adaptive Music Therapy, Intelligent Savannah Therapy	cross-sectional	VR	14	Negative emotions	Significantly less frustration	57	*p* < 0.005 *F* = 7.72
	Anguera et al.	Video game (BBT)	Longitudinal (2 months, 1 year)	IPAD	5(Fz, FPz, AF3, AF4, AFz)	mft power	theta power increases to a level comparable to that of young people	49	*d* = 0.74
	Amjad et al.	Xbox 360 Kinect cognitive games	Longitudinal (6 weeks)	VR	14	slowness and complexity	(1) Increased low-frequency power bands and decreased in high (2) Decrease in EEG complexity (3) Reduced synchronization	44	delta (*p* = 0.013), theta (*p* = 0.002), beta waves (*p* = 0.046), complexity (*p* = 0.016)
	Israsena et al.	Brain training games with NFT	Longitudinal (10 weeks)	PC	16	Alpha and beta rhythms	Improvement in resting (eyes open) upper alpha activity measured from the occipital region	35	*p* = 0.04
	Yang et al.	virtual-reality-based cognitive training (VRCT)	Longitudinal (8 weeks)	VR	19	TBR TAR DAR	The alpha connectivity in DMN region was higher in intervention group than in control group	99	*p* < 0.05
	Styliadis et al.	Brain Fitness	Longitudinal (8 weeks)	computer	\	Delta, Theta, Beta 1, Beta 2	(1) Delta, Theta, and Beta Rhythms significantly decreases; (2) The EEG decrease in PCu/PCC	70	*r* = −0.546
fNIRS	Acevedo et al.	App-based ABC games	Longitudinal (4 weeks)	tablets	PFC	blood flow changes in the FC, the strength of RPFC activity	Increased left DLPFC activity (CT-NF group)	86	Significant (β: 0.26–0.33)
	Liao et al.	Exergaming	Longitudinal (12 weeks)	computer	PFC	HbO-derived concentration changes	(1) Reduced activation of the PC (2) Higher neural efficiency	46	LPFC: small (η^2^ = 0.015) RPFC: small (η^2^ = 0.014)
	Ge et al.	Avoid falling game	cross-sectional	mobile phones and VR	PFC, MC	Degree of brain activation (FC, MC)	(1) Cell phone games:activate FC (2) VR games:activate MC	37	*r* = 0.624 (youth group) *r* = 0.771 (elderly group)
	Vermeij et al.	Computerized WM training program	Longitudinal (5 weeks)	computer	PFC	hemodynamic response in LPFC, RPFC	Stronger increases of HbO2 at baseline	35	Large effect (η^2^ = 0.767)
	Eggenberger et al.	Cognitive-motor video game (DANCE)	Longitudinal (8 weeks)	Impact Dance Platform	PFC	absolute concentrations of HbO2 and Hb	Significantly reduced oxygenation of the left and right hemisphere PFCs	33	Medium to large (*r* = 0.31–0.50)
	Sato et al.	Step Mania 3.9(Dance video game)	Longitudinal (12 weeks)	computer	FPC, DLPFC	HbO2	(1) Significant increase in right DLPFC activation (Stroop CW task) (2) Significant increase in left DLPFC activation (Stroop W task)	21	Large [ES = 1.08 (Stroop W)] Medium [ES = 0.79 (Stroop CW)]
	Yu et al.	Video game-based bilateral upper limb training (VGBULT)	cross-sectional	computer	PFC, MC	HbO2	(1) Stronger activation of MC and PFC (2) Enhanced functional connectivity between PFC and MC	18	MC: *p* < 0.05 PFC: *p* < 0.05
EEG + fNIRS	Zhang et al.	Memory, Executive function	Longitudinal (24 weeks)	tablets	64	EEG: Clustering coefficient and average path length fNIRS: The blood volume change (FC)	Correlation between changes in MRI/EEG/NIRS data and changes in behavioral data	120	*d* = 0.74(working memory) *d* = 0.575(executive function)
	Kober et al.	Spatial navigation task	cross-sectional	VR	32	fNIRS: frontal and parietal brain regions of the right hemisphere	EEG: Significant increase in theta wave power (FC).	27	Moderate effect (η^2^ = 0.60)
	Angsuwatanakul et al.	Visual memory task	cross-sectional	computer	EEG: 30 fNIRS:28	MSE	fNIRS: Significant increase in HbO2 values	15	Medium *r* = 0.39
	Makedon et al.	3D video games	cross-sectional	3D projector	38	EEG: SampEn fNIRS: FC, PC	Brain complexity is higher when remembering deliberately compared to forgetting deliberately (FC, PC)	10	Medium

MSE, multiscale entropy; NFT, neuralfeedback training; mft power, midline frontal theta power; PFC, prefrontal cortex; MC, motor cortex; LPFC, left prefrontal cortex; RPFC, right prefrontal cortex; LMC, left motor cortex; RMC, right motor cortex; TBR, theta/beta ratio; TAR, theta/alpha ratio; DAR, delta/alpha ratio; DLPFC, dorsolateral prefrontal cortex; SampEn, sample entropy; *d*, Cohen’s *d*; η^2^, SSA/SST; *r*, Pearson’s correlation coefficient; β, standardized regression coefficient; CP, central parietal; HbO2, oxyhemoglobin.

## 3 Results

There is a paucity of current research that can quantify the impact of cognitive training and give evidence to support the design of cognitive games. Therefore, researchers have sought multiple neuroimaging techniques, including MRI, EEG (Ricard et al., 2023), positron emission tomography (PET) (Perrin et al., 2009) and fNIRS, among others, to monitor the effects of digital therapy. However, single neuroimaging has limitations in terms of spatial and temporal resolution, and therefore more and more researchers are adopting multimodal techniques to provide information in more spatial and temporal dimensions. The portable and flexible nature of EEG and fNIRS devices compared to traditional invasive or large scanning techniques, as well as the complementary spatiotemporal resolution of EEG and fNIRS (Shin et al., 2018), makes them more suitable for monitoring changes in brain biomarkers in real time while performing digital game training. In this part, we summarized the application of EEG, fNIRS, and multimodal detection in AD digital therapy, and the visualized results were shown in Table 1.

**Figure 3:**
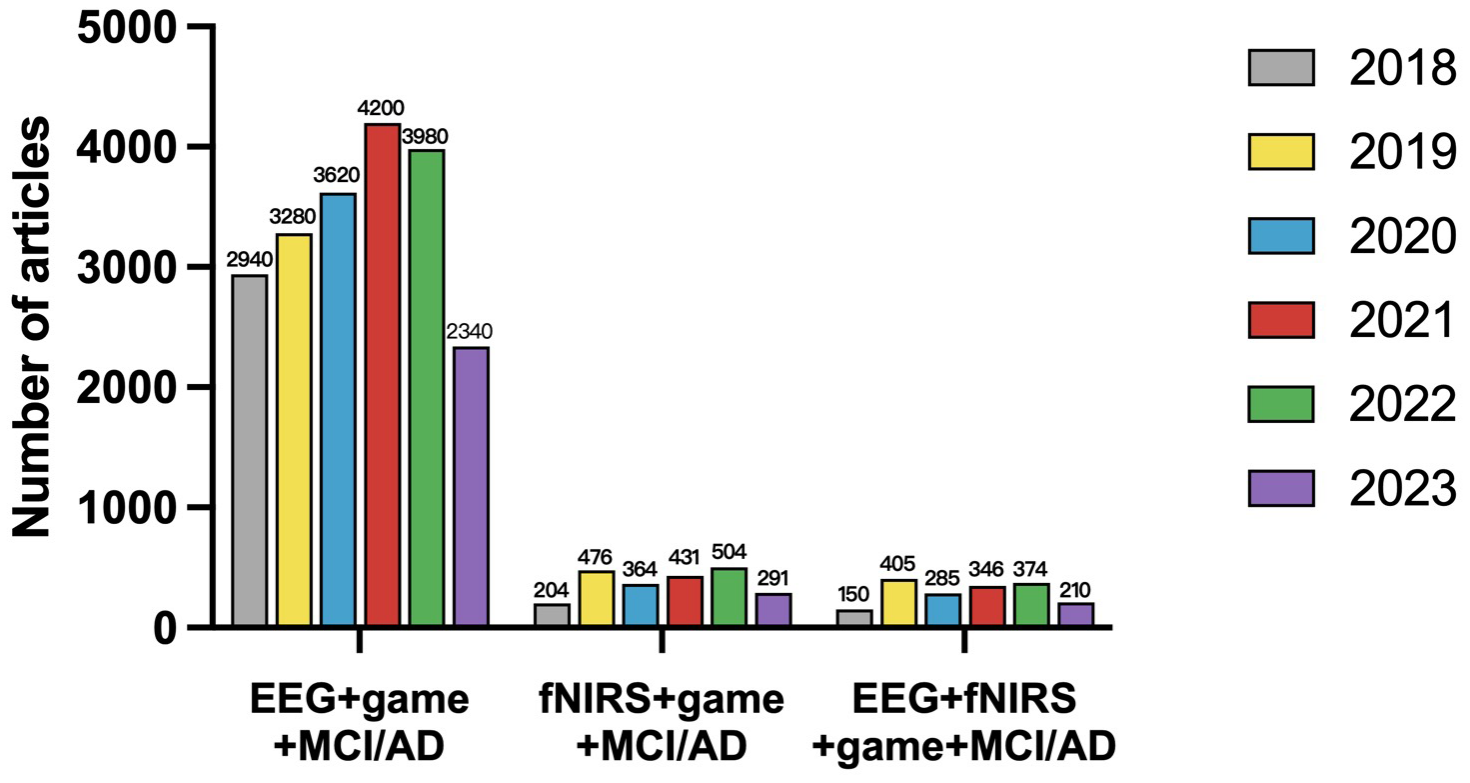
The number of articles published from 2018 to July 2023 on the detection and treatment of cognitive impairments using digital therapeutics with EEG/fNIRS/EEG + fNIRS.

We used Google Scholar to conduct classified quantitative statistics of academic papers on AD digital therapy in the past 5 years using EEG and fNIRS as detection tools. The statistical results are shown in Figure 3. According to Figure 3, the study of neural detection methods increased most in 2018–2019, after which volatility increased. From the proportion analysis of detection means, the application research in the detection means the field is mainly EEG. However, in recent years, multi-modality imaging of fNIRS and EEG + fNIRS is also becoming a new research hotspot. This further validates the value of a systematic review of the three detection modules.

### 3.1 EEG cognitive training in AD

#### 3.1.1 Tablet and laptop-based cognitive therapy

**Figure 4:**
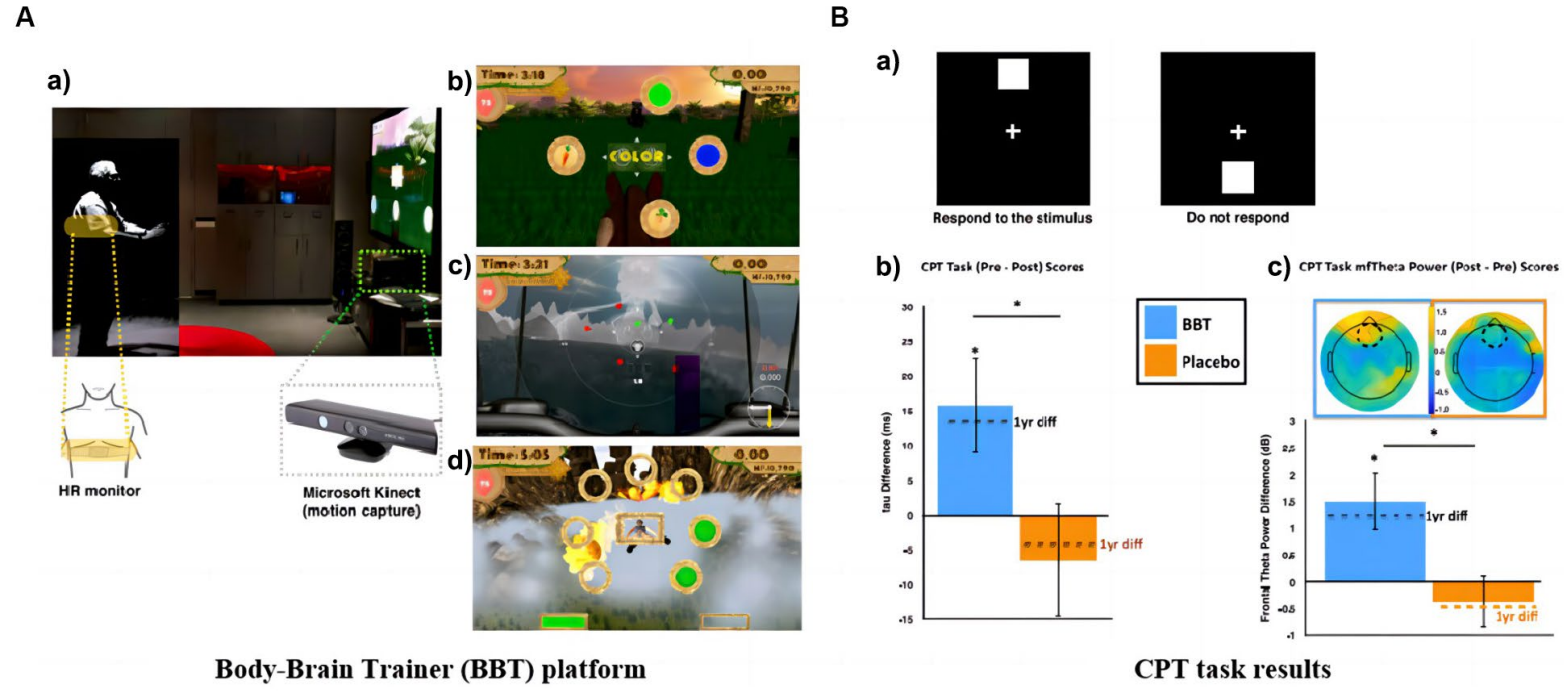
Remote training games: **(A)** BBT platform: **(a)** Microsoft Kinect motion capture technology and real-time intensity adjustment image; **(b)** Task switching module; **(c)** Attention module; **(d)** Working memory module. **(B)** CPT task results: **(a)** Stimuli and protocol for CPT task; **(b)** Between-group mean change in Gaussian tau and change in tau after 1 year; **(c)** Between-group mean change in frontal midline theta power (Anguera et al., 2022).

Declines in working memory and sustained attention are considered to be subtle indicators of cognitive changes before a significant decline. Maintaining attention and the quality of the elder’s life is at stake. Mobile technology for remote training allows more frequent interventions, while portable EEG devices can provide longitudinal assessments of the effects. This is a strategy that facilitates the conservation of time and space resources. Based on this, California researchers created a video game (Body–Brain Trainer: BBT) with an integrated approach to adapt to the cognitive and physical needs of the player to reach some kind of personalization (Anguera et al., 2022). Forty-nine healthy older adults used iPad for 2 months of remote training. BBT treatment effects are monitored in physical health and attention. For attention monitoring, midline frontal theta (MFT) power was selected as a feature. By comparing the monitoring data before and after 2 months and 1 year, it was found that the improvement in attention outcome measures in the BBT group exceeded the performance prediction. The game interface with changes in tau and MFT power is shown in Figure 4.

**Figure 5:**
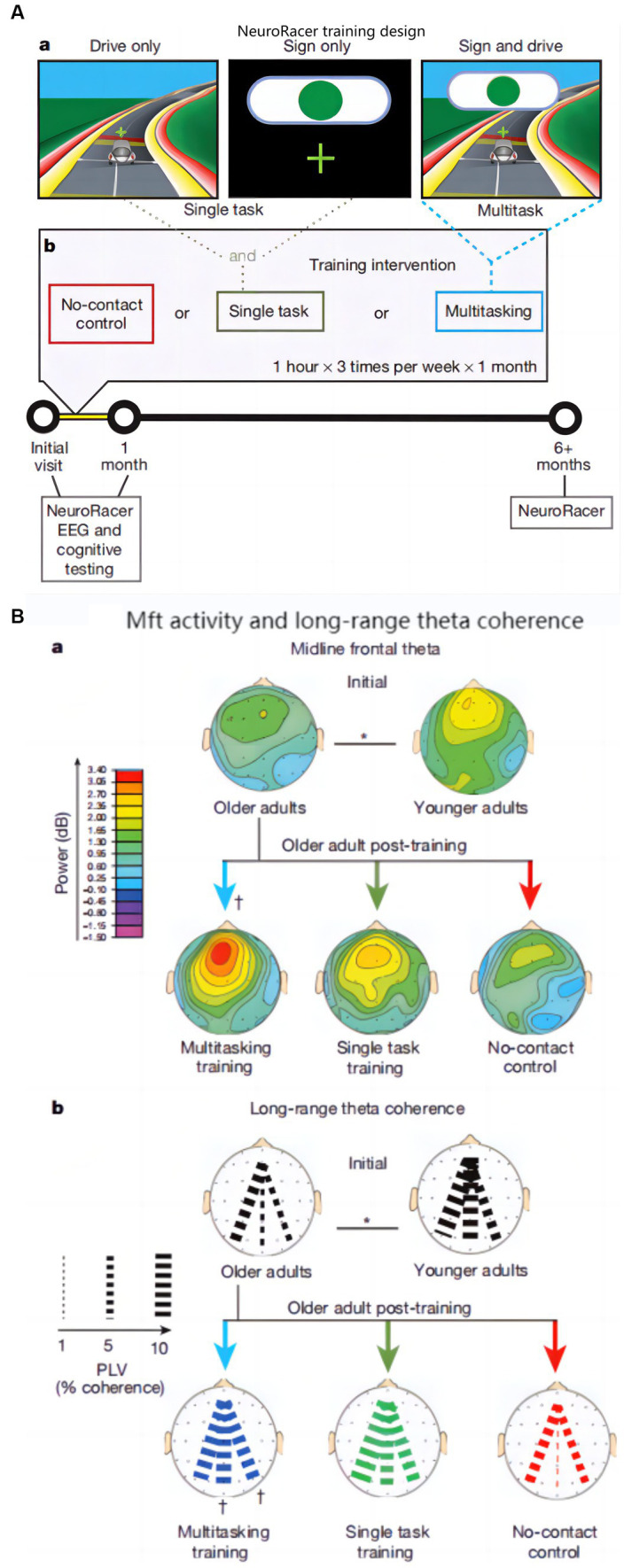
NeuroRacer: **(A)** Experimental conditions and training design: **(a)** Screen shot in various condition, **(b)** Visualization of training design; **(B)** Younger and older adults’ MFT activity and long-range theta coherence: **(a)** MFT activity, **(b)** Long-range theta coherence (Anguera et al., 2013).

The follow-up investigators laterally expanded the age range of the subjects and developed a 3D video game (NeuroRacer) for working and sustained memory, designed as in Figure 5A (Anguera et al., 2013). The ability of MFT was used as a biomarker in the study to evaluate the training effect. After 6 months of training, the effect was still sustained, the results are shown in Figure 5B.

In addition to Anguera’s team, other researchers are trying to combine computer-based digital therapy with EEG to improve older adults’ cognitive abilities. A study by Israsena et al. used EEG neurofeedback techniques combined with brain training games for cognitive training (Israsena et al., 2021). In a 10-week longitudinal study, participants trained on a PC game where in-game elements responded to their attention level, with increased focus leading to faster bear racing, a happier young girl, hotter kitchen fires, quicker paper airplane flights, and improved basketball shooting. Real-time neural feedback adjusted game difficulty to exercise cognitive skills. During training, the researchers focused on changes in Alpha and Beta rhythms to assess the training effects. Jirayucharoensak et al. (2019) also used the same game for a 3-month training period, again focusing on Alpha and Beta band activity. Rose et al. (2015) employed a computer game called Virtual Week to teach older persons cognitive skills, and the training material they chose was more closely connected with daily living. The week in real life is modeled in the game. A range of daily tasks meant to exercise their prospective memory skills were given to them. The researchers measured the training effect during a 1-month longitudinal training period by tracking changes in ERP characteristics like N300, P3, and PP.

Nan et al. found that digital training enhances a person’s alpha band’s relative amplitude and that an increase in short-term memory is positively correlated with an increase in an individual’s upper alpha value (Nan et al., 2012). Researchers from California trained young and older adults using a combined BBT and MBT task, recording data on tau protein, EEG changes, and reflections from other indirect measures after the combined task and 1 year later, respectively (Anguera et al., 2022). It was shown that tau protein levels decreased significantly after training, while MFT power energy increased significantly compared to pre-training. However, the change in reflective speed was not significant for pre- and post-training monitoring using working memory. In contrast, MFT power had a higher sensitivity to improvements in sustained attention abilities and working memory. The study by Israsena et al. focused on changes in Alpha and Beta rhythms. The study found that after a 10-week training period, participants showed improvements in resting-state (eyes open) upper alpha activity measured from the occipital region, suggesting some improvement in cognitive domains (e.g., attention) (Israsena et al., 2021). The study by Rose et al. (2015) in contrast, concentrated on modifications to ERP characteristics such N300, P3, and PP. The study saw an increase in the amplitude of the late positive complex (approximately 900–1,200 ms) in the right frontal electrode cluster, but a decrease in amplitude in both groups at the same time points in the left occipitoparietal electrode cluster. According to Rose et al. (2015), these changes correspond to an anticipated increase in memory capacity. However, not all research on older individuals’ cognitive training discovered altered biomarkers. This work focused on changes in Beta and Alpha band activity without describing in detail the results of biomarker validation of AD (Jirayucharoensak et al., 2019). Overall, these studies suggest that cognitive training based on games and neurofeedback techniques has some positive impacts on mild cognitive impairment and aging adults’ cognitive performance. This conclusion is supported by biomarkers.

Compared with healthy older adults, patients with MCI tend to have a higher theta rhythm (Jelic et al., 1996; Grunwald et al., 2001), while patients with AD mainly have a higher delta rhythm (Dierks et al., 1993; Huang et al., 2000; Babiloni et al., 2004; Jeong, 2004). Furthermore, increases in theta and delta were strongly associated with a decrease in the volume of brain regions associated with bilateral memory circuits in AD patients (Fernández et al., 2003; Grunwald et al., 2007). Styliadis et al. (2015) used Brain Fitness software to conduct cognitive training for MCI patients, combined with physical training, and found that training can significantly reduce delta and theta rhythms. At the same time, the researchers also observed a decrease in the EEG of the Precuneus (PCu)/Posterior Cingulate Cortex (PCC). As a major hub of default mode network (DMN), the decrease of EEG in PCu/PCC indicates adequate function and enhanced plasticity in DMN brain regions (Styliadis et al., 2015).

#### 3.1.2 Novel virtual reality-based systems

Various preclinical AD research are using VR-based, simulation display games, and other promising therapy technologies. EEG can be utilized as a clinical monitoring system to evaluate its efficacy. Imran conducted physical activity and cognitive training in Pakistan using a VR Xbox 360 Kinect platform (Amjad et al., 2019a,b). This study extracts the long-term and complex features of EEG in order to investigate the effects on the cognitive functions of MCI patients both horizontally and longitudinally (Amjad et al., 2019a,b). The researchers use Emotiv Epoc with 14 channels to record EEG features. After 6 weeks, the cognitive function has been improved. To investigate the effects of VR on attention and memory in MCI, Thapa et al. conducted an 8-week study using a VR platform to extract power spectral density ratios to analyze the effect of the intervention (Thapa et al., 2020).

MCI subjects have lower EEG complexity due to neuronal death and decreased neurotransmitter levels, thus using EEG complexity as a biomarker for MCI diagnosis. Studies of cognitive training of older adults with VR games have shown that (Amjad et al., 2019a,b). By analyzing the change in EEG complexity or approximate entropy (ApEn) before and after the cognitive game, an increase in complexity was found, indicating an improvement in cognitive function with the new therapeutic VR game. Thapa et al. found that in the VR intervention group, compared to baseline, at follow-up theta was significantly lower around the parietal and temporal regions. But in the VR intervention group, TBR was lower in the temporal and parietal regions at follow-up, and all power ratios did not alter in the control group (Thapa et al., 2020).

There is evidence that alpha connectivity in the DMN region is decreased in patients with MCI (Brier et al., 2014). However, after Yang et al. conducted virtual-reality-based cognitive training (VRCT) and exercise intervention for patients with MCI, the alpha connectivity in DMN was higher in the intervention group (Yang et al., 2022). This means that the VR game intervention improved cognitive function associated with the default mode network in patients with MCI.

### 3.2 FNIRS cognitive training for AD

#### 3.2.1 Tablet and laptop-based cognitive therapy

**Figure 6:**
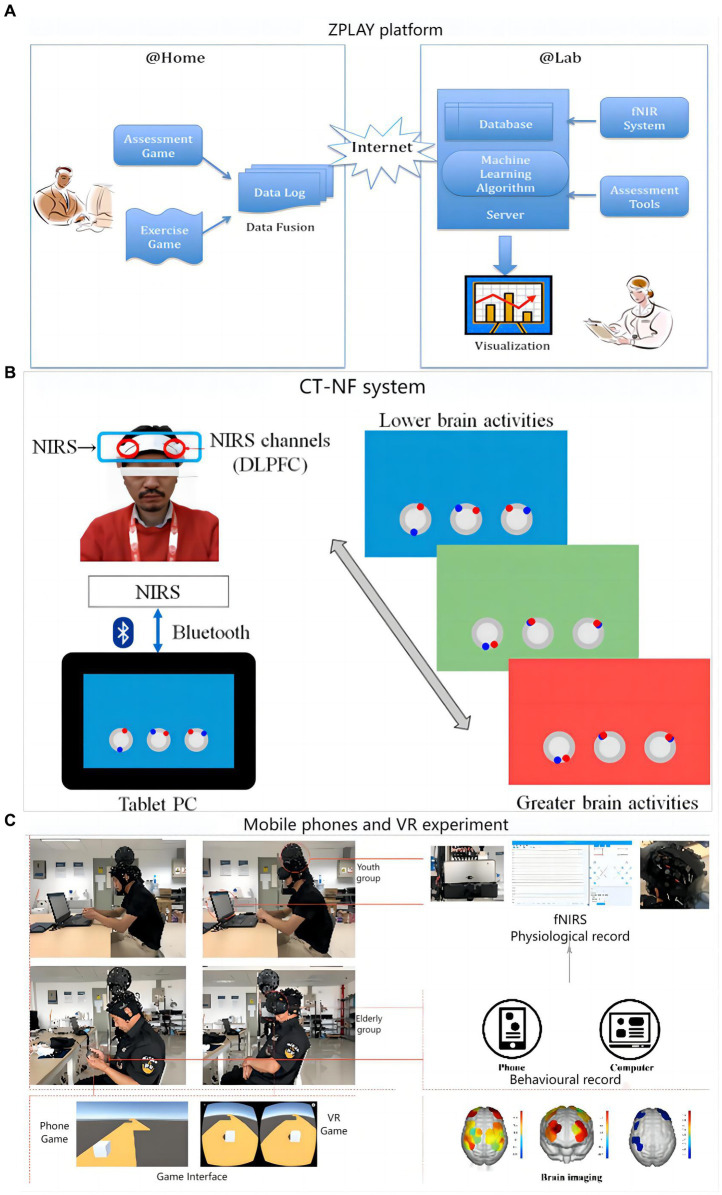
Digital training flow using fNIRS as a medical assessment: **(A)** ZPLAY platform; **(B)** Cognitive training with neurofeedback system; **(C)** Flow chart of the mobile phones and VR experimental set-up.

Cell phones and tablets are a fixture in modern life. The fNIRS based on neural coupling has great potential in training systems. In addition, fNIRS is insensitive to artifacts, more adaptable in different situations, and is a convenient mobile monitoring companion device. Fillia Makedon developed the ZPLAY digital intervention therapy system for AD (shown in Figure 6A), which measures cerebral blood flow before and after cognitive training using a portable fNIRS (Makedon et al., 2010).

A digital treatment study on cognitive training with neurofeedback (CT-NF) was conducted by a team at Tohoku University (Acevedo et al., 2022). Sixty-eight elders were divided into three groups, each performing app-based ABC games on a tablet process as shown in Figure 6B. The training consisted of three dimensions: processing speed, memory and attention. The entire training was done remotely at home for 20 min a day for 4 weeks, and the brain was measured before and after the training using fNIRS. Notably, the researchers also considered incorporating exercise into the digital assessment, including having the older adults dance and move their limbs. In a research by Sato et al. (2023) community-dwelling older persons with MCI received 12 weeks of longitudinal training using the dance computer game Step Mania 3.9. Participants played the dance game on a computer and exercised their cognitive skills by completing the dance moves in the game. The study by Eggenberger et al. used a cognitive-motor-based video game dance (DANCE) for 8 weeks of longitudinal training in older adults (Eggenberger et al., 2016). By completing in-game dance movements and related cognitive tasks, participants who played the game on the Impact Dance Platform exercised their cognitive and balance skills. A study by Liao et al. (2021) compared the effects of Exergaming and combined exercise on cognitive function in frail older adults. In a 12-week longitudinal study, participants performed exergaming training on a computer to exercise their cognitive abilities by completing various cognitive and motor tasks. A study by Yu et al. on cognitive training of older adults through video game-based dual upper limb training (VGBULT) (Yu et al., 2022). Participants played the game on a computer and exercised their cognitive and motor coordination skills by completing the dual upper limb motor tasks in the game. Overall, the combination of games and motor tasks provides a fun and easy-to-adhere approach to cognitive training for MCI sufferers and older individuals. To some extent, these methods have a positive effect on improving cognitive function.

Researchers at Tohoku University focused on blood flow changes in the frontal brain and the strength of right prefrontal cortex (RPFC) activity. They found that DLPFC brain activity was stronger on the left side of the CT-NF group, which was positively correlated with improvements in working memory and concentration. Sato found that after 3 months of dance video game training, the MCI group showed a significant increase in PFC activation during the Stroop task. This indicate cognitive improvement, by examining changes in HbO2 (Sato et al., 2023). Eggenberger discovered that following eight weeks of video dancing instruction, subjects saw a substantial decrease in PFC oxygenation in both the left and right hemispheres during rapid walking, implying an improvement in executive function (Eggenberger et al., 2016). Liao et al. focused on changes in HbO-derived concentrations. After 12 weeks of Exergaming training, reduced activation was found in the prefrontal cortex, thus indicating greater neural efficiency (Liao et al., 2021). According to Yu et al. (2022) after video exercise training, the MC and PFC were engaged, improving functional connection.

We can observe both decreasing and increasing levels of PFC activity, but these results are considered reasonable. According to the works of Gao et al. and Badre et al., this is because the PFC is characterized by hierarchical cognitive control. Depending on the difficulty or familiarity of the task, the activation patterns in different regions of the PFC change. Activation at the mid-dorsolateral PFC (mid-DLPFC) occurs when the task is unfamiliar and difficult, indicating effective training when the activity level increases. As learning and repetition increases, the brain may decrease activity levels in the prefrontal cortex by establishing more efficient short-range fiber connections (Gao et al., 2014). At this point activation occurs primarily at the dorsal premotor (PMd), and a decrease in the level of PFC activity indicates effective training (Badre and Nee, 2018).

#### 3.2.2 Novel virtual reality-based systems

**Figure 7:**
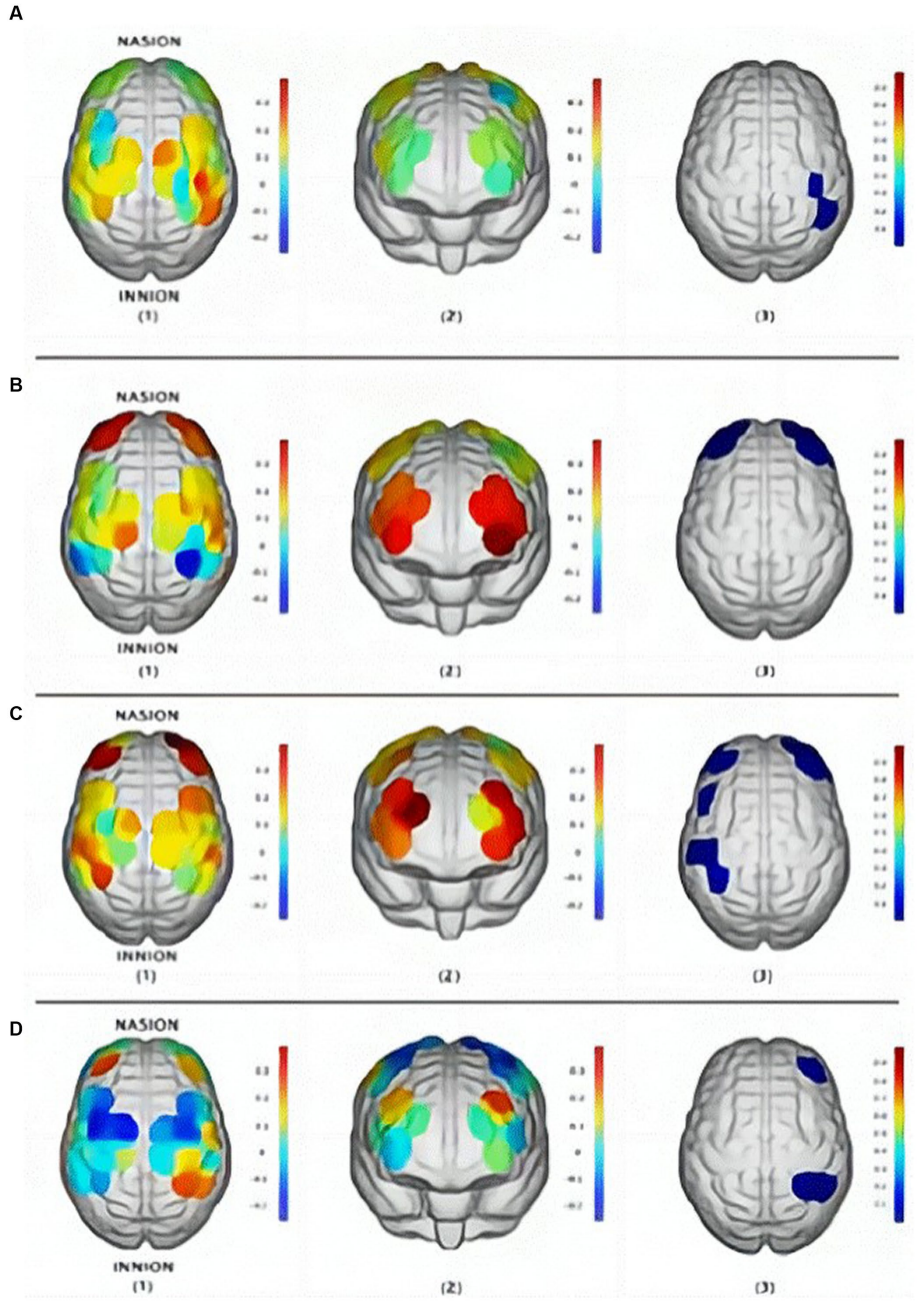
Brain activation states (β change value): **(A)** Youth group in VR games; **(B)** Youth group in mobile games; **(C)** Elderly group in mobile games; **(D)** Elderly group in VR games: (1) motor area, (2) cognitive area, (3) baseline activation (Ge et al., 2021).

A research team in China conducted cognitive training based on cell phones and VR for 37 subjects. During the process, fNIRS was used to monitor the strength of functional connections and signals were collected at PFC and MC (Ge et al., 2021). The overall process and monitoring means are shown in Figure 6C. The findings demonstrated that while VR games significantly more strongly activated the elderly than mobile phone games, mobile phone games more strongly activated the cognitive centers of both groups. This may be because the hand motor function of older adults decreases with age and requires extra effort to move their fingers when using mobile phones, thus activating their motor cortex. Changes in brain activation states before and after mobile phones and VR training are presented in Figure 7.

### 3.3 Synergistic effects of hybrid neuroimaging in cognitive training for AD

#### 3.3.1 Optimized training model

Related studies have shown that nerves are plastic and that the nervous system reorganizes its structure, and functional connections, in response to extrinsic stimuli, resulting in enhanced cognitive abilities (Cramer et al., 2011). Based on this, cognitive training of AD and MCI patients by nonpharmacological means has attracted much attention as a safe and economical way. To assess the training effects and explore the neural correlates behind them, researchers have tried to find an alternative to invasive monitoring techniques to better accommodate digital therapy. During digital therapy, the assessment of neurovascular coupling is more sensitive to the enhancement of cognitive abilities (Dutta et al., 2015). One researcher demonstrated the potential of this multimodal approach with a visual memory task, suggesting that multiscale entropy (MSE) may be a common physiological indicator of EEG and fNIRS. The results suggest that remembering things requires more effort than forgetting them, and therefore exhibits higher activation intensity and higher levels of brain complexity. And more difficult memory tasks are also more likely to stimulate the brain. These results suggest that MSE may be a potential marker for multimodal brain function analysis (Angsuwatanakul et al., 2020). Therefore, the combination of multi-modal physiological indicators and deep learning has become an important monitoring tool to optimize digital training. The specific integration of multimodal testing in practical applications is to look for the relationship between abnormal EEG activity and blood flow in the brain (Jones et al., 2000). AD patients usually show reduced oxygenation levels in certain areas of the brain, which may be associated with impaired cerebrovascular function. This decreased oxygenation level may be associated with abnormal EEG activity such as increased delta waves and decreased alpha waves (Roohi-Azizi et al., 2017). In addition, brain networks in AD usually show abnormal connectivity. EEG can be used to study synchronization and interaction between different regions of the brain, and these changes may be associated with irregular distribution of cerebral blood flow.

**Figure 8:**
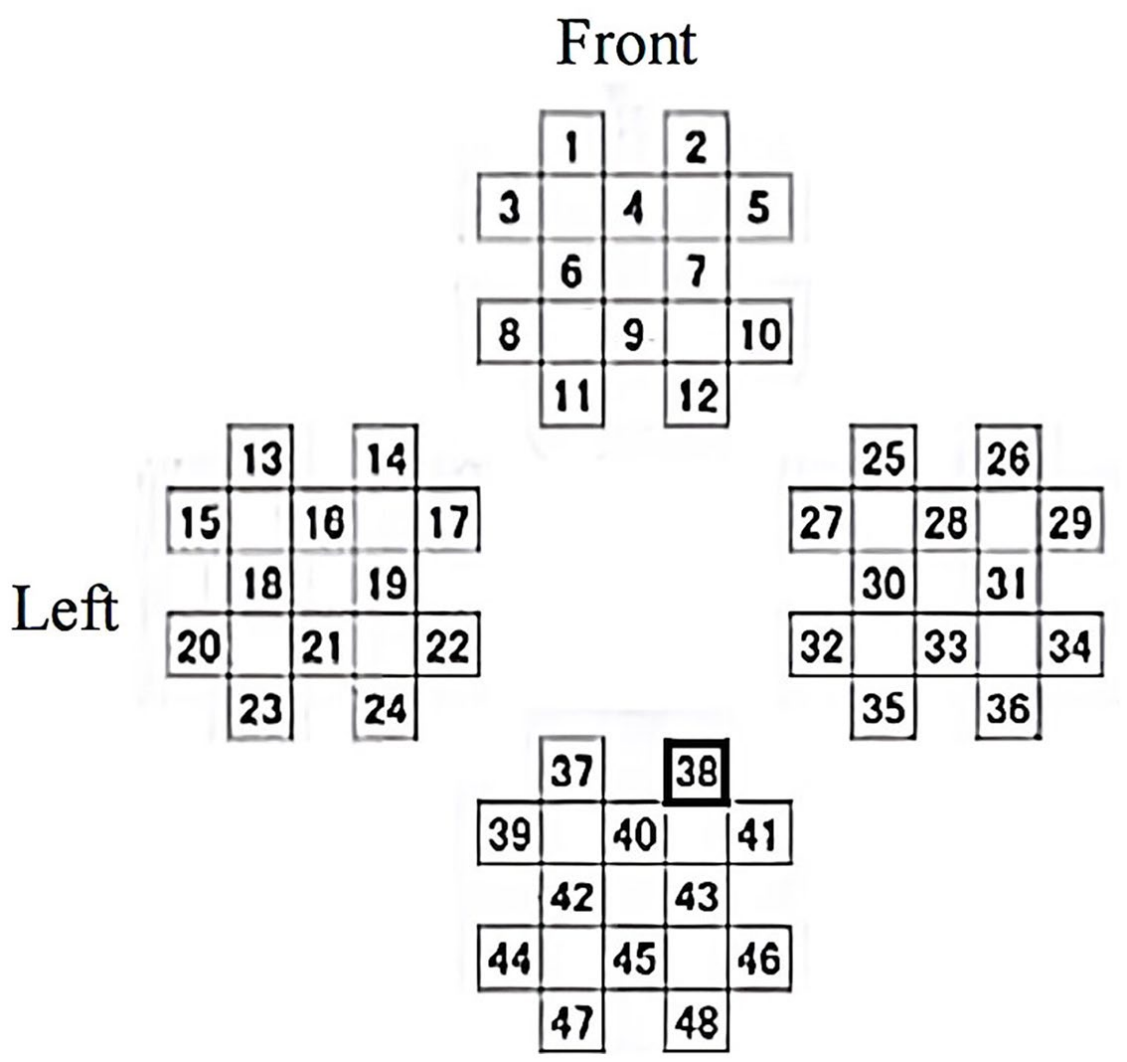
fNIRS measurement channel layout.

The Peking University Institute designed six tasks for 120 MCIs at the dementia center to assess the effects of memory and executive training on cognitive performance. Features include aggregation coefficient, mean path length, and changes in frontal lobe blood volume (Zhang et al., 2018). David compared sample entropy changes after the early AD and healthy control baseline and WM tasks, using whole-brain EEG and fNIRS in the FC. The results showed that multimodal EEG-fNIRS complexity analysis can detect WM decline, which may provide new clues for treatment (Perpetuini et al., 2020). Researchers from Japan explored the effects of 3D video games, using cerebral blood flow as a bio-indicator to measure changes in brain activity before and after baseline. FNIRS locations are shown in Figure 8, and analyzing eye movements in conjunction with EEG to confirm that there is no peripheral vision influence when viewing images (Takada et al., 2018).

In summary, multimodal medical assessment using EEG and fNIRS allows exploring the tasks that stimulate the greatest neuroplasticity during digital therapy, thus optimizing training modalities.

#### 3.3.2 Personalized treatment plan with multimodal monitoring

Digital cognitive therapy allows for individualized treatment plans based on the patient’s cognitive abilities and needs, resulting in more effective treatment outcomes. In addition, brain function recovery is better when medication and daily exercise are added to digital training (Manchanda et al., 2022). The use of VR in neurological rehabilitation is receiving increasing attention (Rose et al., 2005). VR games offer the opportunity to develop specific environments that can be tailored and become useful tools for rehabilitation. VR can create personalized natural environments that improve training validity and learning generalization (Schultheis and Rizzo, 2001). Another advantage is the economy of scale, where the same VR technology can be applied to many types of exercises and many types of patients. Therefore, VR can be used for virtual navigation training for patients with neurological diseases, and it can also be used to treat desensitization for patients with sexual phobia (Kober et al., 2013). Non-invasive fNIRS technology is an emerging real-time imaging tool that is highly resistant to interference. Therefore, the key to combining fNIRS with VR is that fNIRS measurements do not limit immersion, and motor behavior during VR produces fewer data artifacts. At the same time, combining with EEG to obtain finer temporal resolution and deeper localization tracking opens up the possibility of studying the underlying cortical electrical processes.

#### 3.3.3 Multimodal neurofeedback

The combination of BCI and behavioral physical therapy is a viable option for cognitive rehabilitation, capable of restoring lost function by inducing neuroplasticity. This, in turn, requires extensive clinical trials using invasive and non-invasive BCI with long-term follow-up of patients (Chaudhary et al., 2016). However, invasive BCI requires special equipment, cumbersome procedures, and high safety risks. In contrast, digital cognitive therapy uses mobile devices, is easy to perform, and has no significant safety risks. And it is performed at home, with low treatment costs, long duration, and a more flexible format. A remote home therapy platform is proposed to facilitate the sharing of data in real time with therapists on the cloud. This approach centers on attention and motor imagery, recording EEG, EMG, and other data as assessments (Rahman et al., 2019). Carlo Fantozzi built an Informa Software Platform for managing patients with neurocognitive disorders (NCD). NCD users can access more than 20 serious games at home. Therapists can customize activities, assign them to patients, and collect telemetry data to monitor progress through the platform program (Fantozzi et al., 2022). Part of the digital platform also supports monitoring systems to provide safety detection and tracking activities, monitoring the patient’s physical condition with transparent and objective data storage (Vergara et al., 2015). Thus, in the case of teletherapy with regular monitoring, patients can continue their recovery.

## 4 Discussion

### 4.1 Experimental design

In terms of the sample, the small sample size is a major limitation (Amjad et al., 2019a,b; Israsena et al., 2021; Yu et al., 2022; Sato et al., 2023). From the perspective of subjects, one of the reasons is that subjects quit midway (Amjad et al., 2019a,b). However, the main reason may be that the number of target subjects themselves and their ability to participate in the study have higher requirements for recruitment conditions (Yu et al., 2022), resulting in greater recruitment difficulty (Sato et al., 2023). The difficulty of building a suitable experimental environment also makes obtaining large samples impractical. Insufficient sample size may limit the effect size that can be achieved in a study, and too small an effect size increases the probability of false-positive and false-negative results (Israsena et al., 2021). Another limitation of the sample is the bias like the sample, with an imbalance in the proportion of men and women (Thapa et al., 2020), single-sex structure (Jirayucharoensak et al., 2019), and imbalance in the age ratio (Delisle-Rodriguez et al., 2023) which would limit the explanatory power and generalizability of the study results, and also lead to the variation brought by gender and age being difficult to be explored. Future studies should increase the sample size and balance the age and gender distribution of subjects. Researchers can consider more recruitment channels to increase the sample size of the study, and control and screen the age and gender distribution of the subjects in advance. At the same time, researchers also need to make efforts in game development, improve the playability and attractiveness of games, attract participants, and reduce the turnover rate of participants (Thapa et al., 2020). For intervention studies, whether they are pharmacological or psychological interventions or any other form of intervention, the role of a blank control group beans is crucial to exclude confounding factors in intervention experiments, determine whether the experimental results are affected by the intervention treatment, and improve the reliability and accuracy of the experimental results. However, there are still some studies lacking a blank control group in digital training (Eggenberger et al., 2016; Liao et al., 2021), the reason behind this may be the limited time and cost, but it is not a difficult issue to take into account from the idea of experimental design, and therefore it is a more difficult limitation to tolerate. Future studies should set up a blank control group, meaning that this group of subjects received no intervention, while the other group of subjects received a digital intervention, and finally compared the cognitive function of the two groups. This will help to test the effect of digital training more reliably and accurately. Psychological attributes are different from physiological attributes, and their universality is relatively weak, so individual differences at the psychological level are much larger than those at the physiological level. And due to technical limitations, current digital interventions are often standardized rather than individualized, so it is difficult to take care of individual differences and individual needs of subjects in motivation, learning strategies and responsiveness, preferences, etc. (Anguera et al., 2013). It may be a feasible idea to combine digital therapy with neurofeedback technology to allow digital therapy to perform self-adjusting interventions based on the subject’s personalized neurofeedback (Israsena et al., 2021). Of course, technologists need to improve the personalized ability of digital intervention technology by improving technology, in order to achieve the “right medicine” effect. If this idea is fully realized, digital treatment devices will be able to approach human doctors who provide consultation services in hospitals.

### 4.2 Digital training

#### 4.2.1 Characteristics of the elderly

The content and modality of digital training is a relatively unfamiliar thing to dementia patients, and dementia patients will show a systematic defensive posture in the face of anything unfamiliar, so dementia patients’ defensiveness may already be relatively strong in itself when facing digital interventions (Benveniste et al., 2012). In addition, digital training games may also bring about negative emotions in dementia patients, such as the visual instructions used in the game related to abstract concepts that often confuse them, and the complexity of the system that brings about anxiety and fear (Benveniste et al., 2012). This suggests the need for a simple and enjoyable aesthetic design for number games and enhanced flexibility in task difficulty adjustment, which would not only help to improve the game experience for people with dementia but also to attract subjects other than those with psychosis, thus expanding the sample size (Benveniste et al., 2012). At the same time, to detect subjects’ adverse emotional experiences in time to adjust the training task, it is also necessary to combine with neuromonitoring techniques that can provide real-time feedback to determine subjects’ current task status through real-time changes in biomarkers. In addition to psychological discomfort, some of the movement-related digital treatments may also cause more serious harm in terms of physiological damage. For example, digital interventions related to dance and physical movement require subjects to perform a certain level of exercise (Eggenberger et al., 2016; Amjad et al., 2019a,b; Sato et al., 2023), and exercise may impair the physical health of older adults with cardiovascular disease. This suggests that researchers not only need to do neuropsychological measurements before the start of the experiment, but also need to conduct targeted investigations and strict screening of the physiological health status of the subjects, such as cardiovascular disease screening, to prevent additional damage in the subjects with unsuitable physiological conditions.

#### 4.2.2 Limitations of the game itself

Due to the relatively late start of digital therapy and the commercial interest in technology development, there are currently fewer types of digital intervention games for the elderly than games developed for the general population (Anguera et al., 2013). In the future, researchers not only need to enrich the types of games suitable for the elderly from the level of technical development, improve the measurement breadth, but also need to combine neural monitoring technology to obtain more types of data, such as EEG, heart rate and other types of data. At the same time, researchers also need to explore the commercial value of digital intervention games and attract commercial capital to enter, because capital is a prerequisite for all development ideas to be implemented. The device itself can also bring discomfort to the subject, such as VR devices may bring motion sickness, headache, eye strain, and other discomforts (Rebenitsch and Owen, 2016; Abdessalem et al., 2021). In the future, researchers need to combine the neurofeedback and subjective reports of the subjects to optimize and adjust the parameters of factors that may cause discomfort such as the complexity of the game environment and the speed of movement. In addition, researchers need to give the subjects enough rest time during the experiment, so that the physiological and psychological state of the subjects can get a buffer. In terms of the environment in which the digital game itself operates, although the “digital” nature of the digital game opens up the possibility of remote intervention, there are corresponding confounding variables for remote intervention at home, such as the supervision of the researcher, the location of the treatment, the warm-up (Anguera et al., 2013). The reason behind this may be that self-monitoring by subjects is not a realistic approach, and that remote interventions do not allow for control over the location of the intervention, which may be done in any conceivable location. These several confounding variables are more problematic and difficult to solve. Perhaps a combination of remote and clinical interventions is one way to improve. Patients are allowed to perform mechanical and standardized self-interventions remotely, but are also required to visit the clinic for critical and easily disturbed interventions.

#### 4.2.3 Effectiveness of digital training

As a result, the results of the cross-sectional persistence comparison were limited. These results did not provide a significant amount of data. This lack of data was in regard to demonstrating the superiority of digital training modality efficacy. This comparison was made in relation to other non-digital training modalities. One contributing factor to the limitation was the absence of an active control group (Zhang et al., 2018; Bojan et al., 2021). Moreover, digital training did not exhibit significant distant migration effects compared to other non-digital training (Nouchi et al., 2012). This implies that the digital training effect is currently limited to near transfer effects and does not improve other cognitive functions other than working memory, such as learning, comprehension, and intelligence. Notably, these studies were limited to laboratory settings and did not assess real-world tasks or everyday cognitive abilities (Nouchi et al., 2012; Zhang et al., 2018). It is this limitation that has led to difficulties in the practical application of digital training. In the future, there is a need to establish more cross-sectional positive controls. And researchers also need to evaluate and study the effects of long-distance transfer outside the laboratory, such as affective cognition, interpersonal skills, and so on. These comparisons are useful for exploring the potential of digital training in practical applications. Some studies were limited to cross-sectional studies and lacked long follow-up assessments (Liao et al., 2021; Acevedo et al., 2022; Sato et al., 2023). Thus longitudinally, another issue in the effectiveness is the sustainability of validation through long-term studies (Israsena et al., 2021). It is significant to notice that not only is the distinction between short-term training and long-term follow-up effects investigated here, but also the durability of effects. Studies have shown that short-term training effects have a specific cyclical peak 4 weeks ago, but weaken at long-term follow-up (Acevedo et al., 2022). This is determined by the game paradigm and the pathological characteristics of AD. Therefore, in the future, appropriate training cycles could be established. For example, four times a week at the beginning, and then adjust as appropriate as the condition improves. In addition, because of the possibility of relapse, continuous training and consolidation should not be stopped. These are the keys to optimizing the effect of training. The medications taken by the subjects as confounding factors may also have an impact on the training effects. For example, drug interactions and drug confounding effects may interfere with training outcomes (Amjad et al., 2019a,b). In the future, these factors need to be considered and controlled for in studies to ensure accurate and reliable conclusions are drawn. However, drug discontinuation is a risky behavior for some patients, which leads to a narrow sample selection. Finally, the findings suggest that the laboratory environment is more conducive to the demonstration of training effects than the home environment. Many researchers underestimate the potential benefits of training (Olfers and Band, 2018). Therefore, when designing and implementing training programs, the physical and psychological inconsistencies between the laboratory environment and the home environment should be taken into account. Efforts should also be made to promote and apply the results of the training in real-life situations, and to identify specific problems and obtain directions for optimization through user feedback.

#### 4.2.4 The absence of a generalizable quantitative model

Without the use of neuroimaging monitoring such as EEG, there are quantitative limitations to digital training. In the context of drug metabolism, the lack of physiological markers would make it difficult for researchers to accurately assess the physiological responses and effects associated with brain plasticity (Arshad et al., 2021) and can only be used as a criterion for exposure through time (Manchanda et al., 2022). In terms of pharmacodynamics, the uncertainty of the mechanism of action is a challenge. The rules of digital therapy lack pharmacological guidance and rely more on empiricism (Manchanda et al., 2022). This limitation prevents a comprehensive understanding of the digital treatment cycle and makes it difficult to identify generic models for training dissemination. In this case, the introduction of EEG/fNIRS imaging can be assessed with the extraction of quantitative data on explicit biomarkers. This helps to address the lack of quantitative assessment of drug governance. However, the challenge of identifying generalizable patterns remains, and the neuromonitoring technique itself suffers from interfering signals, as described in the next section.

### 4.3 Psychophysiological and neurological challenges

#### 4.3.1 Challenges of EEG monitoring

First, the quality of the EEG signal is affected by many factors during digital training. During game training, players perform hand, eye, hand, and even whole-body movements, and virtual reality has dynamic and complex environmental factors that introduce biological and environmental noise (Anguera et al., 2013), such as eye-movement artifacts, interfering with EEG signals, which may lead to signal distortion and thus affect the accurate assessment of training effects. To address this issue, researchers need to develop higher-quality EEG devices and employ more advanced techniques such as independent component analysis (ICA) and blind source separation (BSS) during data processing to eliminate noise and artifacts. It is worth noting that eye movements not only produce artifacts to degrade signal quality, but also may interfere with the interpretation of the source of the intervention effect because eye movements produce eye electricity. To address this issue, vertical eye electricity, and horizontal eye electricity need to be calculated and statistical tests are used to demonstrate that there is no significant difference in eye electricity before and after training, thus ruling out that the training effect is due to the effect of eye electricity (Anguera et al., 2013). Second, some of the null results of the current ERP may be due to practical problems with the data collected; Olfers et al. collected only a sample of young people in their study of the intervention effects of video games because the percentage of errors in young people was low, and this may be an important reason why they did not find an effect of training on error-related negative Ne/ERN. This once again suggests the importance of studies collecting a sufficient number and appropriately structured subjects (Olfers and Band, 2018). Third, EEG suffers from a disadvantage in spatial resolution. Due to the presence of the skull, the electrical signal must pass through the skull to reach the EEG electrodes, which leads to attenuation and distortion of the signal, thus reducing the spatial resolution of EEG (Michel and Murray, 2012). This limitation makes EEG potentially less accurate than other neuroimaging techniques (e.g., fNIRS) for studying cognitive processes involving the activity of specific brain regions. Researchers can consider combining EEG with high spatial resolution technologies such as fNIRS to make up for the lack of spatial resolution in EEG, and can also consider using more advanced source localization algorithms to improve EEG localization accuracy (Grech et al., 2008).

#### 4.3.2 Challenges of fNIRS monitoring

The fNIRS is highly subject-friendly, insensitive to fake-movement and low-level movement trajectories, highly robust to dynamic body movements, and can effectively analyze brain function in a variety of motor tasks (Eggenberger et al., 2016; Azman et al., 2017; Ge et al., 2021). However, fNIRS has limitations in assessing deep brain regions involved in motor planning and memory. It can only measure hemoglobin changes near the brain’s surface, potentially missing important neural correlates associated with action tasks (Eggenberger et al., 2016; Ge et al., 2021; Acevedo et al., 2022). Regions like the hippocampus and internal olfactory areas cannot be directly observed (Karim et al., 2012; Kober et al., 2013; Acevedo et al., 2022). However, fNIRS can detect activation-memory links in certain cortical regions, allowing the study of cognitive function and cerebral hemodynamic changes. Increasing activation signal-to-noise ratios can improve results in the future (Kober et al., 2013). Secondly, individual differences in scalp and skull anatomy and changes in the optical properties of scalp tissue can affect the quality and reliability of fNIRS signals. Obtaining consistent signals across participants is challenging (Holper et al., 2010; Acevedo et al., 2022). However, similar EEG and hemodynamic changes may be induced by nonspecific involvement, learning, or stimulus effects. Special care is therefore needed to distinguish whether changes are induced by the subjects themselves or by the training stimuli. Multiple measurements are needed to ensure proper coverage of the desired brain region. Avg. activity is used for analysis. In the future, spatial resolution limitations can be addressed by using bundled photo polar configuration (Ghafoor et al., 2019). FNIRS measures a limited number of channels and typically focuses on task-related brain regions, potentially disregarding important data on motor training adaptations and other brain regions (Eggenberger et al., 2016). To enhance the assessment of functional connectivity, future studies may involve combining frontal regions with measurements of the whole head. More fNIRS channels could be enabled to explore the correlations between different brain regions. The need to expand the probed brain regions is also due to the fact that cognitive decline in Alzheimer’s patients is reflected in reduced signaling complexity at DMN nodes (Chen et al., 2020). In addition, hybrid imaging techniques with EEG, MRI could also be investigated (Ghafoor et al., 2019).

Researchers are concerned about fNIRS artifacts caused by different training methods. Failure to address these artifacts can lead to inaccurate monitoring results. It is important to record EMG data to exclude muscle activity and avoid noise produced by alterations in blood flow brought on by extraocular and frontal muscle activation. Inadequate noise removal may mask true nonlinearity, preventing the observation of statistically significant differences at higher cutoff frequencies (Holper et al., 2010; Takada et al., 2018). Physical artifact removal could enhance the visibility of nonlinearity in CBF variations in the future. Motion artifacts in fNIRS are caused by the movement of the optical poles on the scalp, which is more prominent in VR environments due to frequent head rotation (Holper et al., 2010; Kober et al., 2013; Ge et al., 2021). This can result in motion artifacts. Increases in heart rate and average arterial pressure during tasks can interfere with fNIRS studies, as cerebral blood flow remains stable while skin blood flow fluctuates. Task-induced changes in skin blood flow may mask actual changes in brain activity, requiring caution when analyzing and interpreting data. More study is required to comprehend fNIRS system artifacts and distinguish between extracranial and brain signals (Vermeij et al., 2017). Visual artifacts in SSVEP paradigm games are caused by changes in cerebral blood flow, affecting hemoglobin and oxyhemoglobin levels. Certain video games exacerbate these artifacts with shorter visual stimuli and increased flickering lights (Eggenberger et al., 2016; Takada et al., 2018; Angsuwatanakul et al., 2020; Sato et al., 2023). To reduce their impact, stimulus intensity can be reduced, intervals between stimuli can be increased, background noise can be added, and visual stimuli can be optimized.

#### 4.3.3 Challenges of hybrid neuroimaging

Although the technique using EEG in combination with fNIRS has the advantage of compensating for spatio-temporal limitations. However, based on the goal of reducing mutual interference of pressure between surface sensors, researchers face the challenge of finding the optimal placement of EEG electrodes and fNIRS photoprobes (Chiarelli et al., 2020). In the future, when conducting experiments, multiple ways of combining positions can be tried, depending on the research question, the subject, and the availability of equipment. For example, overlapping layout. Simultaneous placement of EEG electrodes and fNIRS detectors at the same scalp location for tighter spatio-temporal correspondence. Complementary distribution can also be performed to cover different brain regions in order to explore the functional connectivity between different regions. The new incremental layout is no less flexible. In this approach, one technique is first used to measure and then the layout of the latter detector is adjusted according to the lack of information. Additionally, it takes more brain-like algorithms to understand biomarkers and extensive multimodal data processing and integration to extract useful information about brain activity and cognitive processes in AD patients (Angsuwatanakul et al., 2020; Cicalese et al., 2020; Perpetuini et al., 2020). Therefore, the update of signal processing algorithms is necessary. Existing techniques optimize the combination of CNN, LSTM and other deep learning models to form various evaluation models, but some of the accuracy reaches a bottleneck (Chiarelli et al., 2018; Bi and Wang, 2019). In the future, embedding biological models into algorithms can be a breakthrough in algorithmic innovation. This leads to a closer approximation of the real situation and reduces errors. Finally, mixed-modality data, while providing more valid information, also produce more mixed artifacts. Finding the effects of different types of artifacts and removing the artifacts is also a challenge for obtaining reliable brain signals (Ferrari and Quaresima, 2012; Hadjiaros et al., 2023). In the future studies, on the one hand, the artifacts that may be generated in the experimental design should be minimized from the origin. On the other hand, based on the fact that EEG and fNIRS artifacts are generated for different reasons, which originate from the interference received by electrical and optical signals, respectively. Therefore, the techniques for distinguishing and eliminating the artifacts can be improved.

### 4.4 Other challenges

Compared with other factors such as physiology and environment, psychological factors may be the most unstable and difficult to observe and control, but the interference of psychological factors on treatment results can not be ignored. First, emotions can interfere with neuroimaging data. In the process of digital therapy, if the subjects feel anxious and depressed about their cognitive problems, because the withdrawal motivation (Sutton and Davidson, 1997) and negative emotions (Schaffer et al., 1983; Tomarken et al., 1992; Jacobs and Snyder, 1996) are related to the weakening of α power in the right prefrontal lobe, these negative emotions may lead to the weakening of α power in the right prefrontal lobe, thus interfering with the EEG signals of the intervention results. Similarly, negative emotions such as anxiety and depression may also cause changes in blood supply to the brain, which can affect oxygenation levels in brain regions, which can obscure or mask the true effects of digital interventions. One of the important reasons that subjects have negative emotions during treatment is low motivation, that is, low willingness to participate. Low motivation not only led to negative emotions such as irritability about the intervention task, but also reduced the participants’ attention level, ultimately leading to lower performance on the task, which in turn affected EEG and fNIRS signals about attention and other cognitive indicators.

## 5 Conclusion

This systematic review emphasizes the use of neuroimaging in evaluating the effects of digital interventions. Digital therapy shows potential for treating cognitive disorders like AD or MCI, offering individualized treatment, minimal side effects, and economic convenience. By incorporating EEG and fNIRS neuroimaging tools to assess training effects, digital play therapy holds promise for clinical trials. Several novel digital treatments are currently being developed and validated. The review also addresses challenges in experimental design, implementation, and promotion of digital training, as well as enhancing neuroimaging techniques. Valid recommendations and research directions are provided for promoting digital treatment for AD patients. Admittedly, this review has the following limitations. First, this review focuses on biomarker changes in the prefrontal region, but other neural circuits also need attention. For example, the default mode network and working memory related brain regions are associated with attention deficit disorder in older adults and are also worth monitoring. Second, although this review provides a more detailed description of the various challenges in the digital therapy process and gives corresponding recommendations, there are still some uncertainties that cannot be definitively answered: Uncertainties about the clinical efficacy of digital therapy, study design flaws, specificity of reported neurophysiological changes, methodological confusion, and opportunities to advance brain monitoring during treatment are issues that will need to be confronted and addressed in subsequent research. Future considerations include adding a cross-sectional active control group, maintaining a balanced experimental sample, considering the specificity of elderly patients and content diversity in digital treatment settings, and implementing follow-up programs to test the durability effect. In addition, appropriate data processing methods should be selected according to the paradigm characteristics to overcome the limitations of spatio-temporal resolution and to eliminate disturbing artifacts in the experiments. In future technologies, special attention should be paid to the combination of data features, as well as to the choice of spatio-temporal modeling, in order to improve the monitoring of the brain during treatment.

## Author contributions

YCZ: Investigation, Visualization, Writing – original draft. YZ: Investigation, Visualization, Writing – original draft. ZJ: Investigation, Writing – original draft. MX: Supervision, Validation, Writing – original draft. KQ: Supervision, Validation, Writing – review & editing.
